# Anti-Idiotype Vaccine Provides Protective Immunity Against *Vibrio Harveyi* in Grouper (*Epinephelus Coioides*)

**DOI:** 10.3390/vaccines7040210

**Published:** 2019-12-09

**Authors:** Wan-Ling Huang, Shu-Chun Chuang, Chung-Da Yang

**Affiliations:** 1 Graduate Institute of Animal Vaccine Technology, College of Veterinary Medicine, National Pingtung University of Science and Technology, Pingtung 912, Taiwan; e09011lisa@yahoo.com.tw; 2 Orthopaedic Research Center and Department of Physiology, College of Medicine, Kaohsiung Medical University, Kaohsiung 807, Taiwan; f86225016@ntu.edu.tw

**Keywords:** *Vibrio harveyi*, idiotype (Id), internal image, anti-Id vaccine, anti-Id antibodies, anti-Id IgG (Fab)

## Abstract

Since anti-idiotype antibodies (anti-Id Abs) can display internal images similar to the epitopes of the original antigens, we aimed to produce an effective vaccine based on anti-Id Abs to protect grouper from *Vibrio harveyi*. Anti-Id IgG showing *V. harveyi*-like internal images was produced from rabbits immunized with the Id portion of grouper anti-*V. harveyi* antibodies and its Fab portion, anti-Id IgG (Fab), was then prepared to use as the anti-Id vaccine. The resulting anti-Id IgG (Fab) was intraperitoneally injected twice at a 21-day interval into grouper to evaluate its ability to induce effective anti-*V. harveyi* immunity and protection, in comparison with inactivated *V. harveyi* bacteria. We found that administration of grouper with anti-Id IgG (Fab) resulted in enhanced *V. harveyi*-specific serum titers, as well as lymphocyte proliferation. In addition, three weeks after boosting, 90% (18/20) of fish immunized with anti-Id IgG (Fab) survived at least 28 days after a lethal challenge of the heterologous, virulent strain of *V. harveyi*. The capability of this anti-Id IgG (Fab) to imitate the epitopes of *V. harveyi* antigens and effectively induce protective immunity would be advantageous for its application in developing an efficacious vaccine against *V. harveyi* for future farm use in fish.

## 1. Introduction

Orange-spotted grouper (*Epinephelus coioides*), a farmed fish species with a high commercial value, has rapidly become an important profitable agricultural product in Southeast Asia, including Taiwan and China [1]. However, the high intensification of grouper farming is usually accompanied by outbreaks of pathogenic diseases, such as vibriosis, causing dramatic reduction in grouper production [2]. *Vibrio harveyi*, a Gram-negative bacterium, is the major etiological agent of vibriosis in grouper to usually cause deep skin lesions and ulcers, gastroenteric disorders, and eye lesions [3], thereby resulting in high morbidity and mortality in grouper and inducing a significant economic loss [2]. Although antibiotics have been used to control vibriosis in grouper, they result in severe consequences, including the emergence of resistant bacteria and drug residual issues [4]. Vaccination is an alternative option that can significantly reduce such a disease-related threat and antibiotic use [5]. 

Recently, subunit vaccines against *V. harveyi* based on DNA engineering technology have been studied [6,7,8,9,10]. However, *V. harveyi* has been demonstrated to employ multiple critical antigens to maintain its infection in grouper [11]. If broad epitopes of these antigens are simultaneously presented to the immune system, successful immunity and protection against infection may be achieved [12,13]. Therefore, immunization with an anti-*V. harveyi* vaccine containing comprehensive epitopes that can induce strong integral immunity to a broad array of *V. harveyi* antigens is likely to be more effective. Vaccines based on inactivated (killed) bacteria are currently the most commonly used aquatic animal vaccines that can display diverse bacterial antigens [14]. However, the harsh activation processes, such as treatment of bacteria with formalin, heat, or ultraviolet light, induce the denaturation of bacterial immunogenic epitopes or conformations, thereby resulting in impaired function and non-efficient immune responses [15]. More effort is therefore needed to improve not only the immunogenic epitope integrity presented by vaccines, but also the immune responses and protection they induce in animals. 

**Figure 1:**
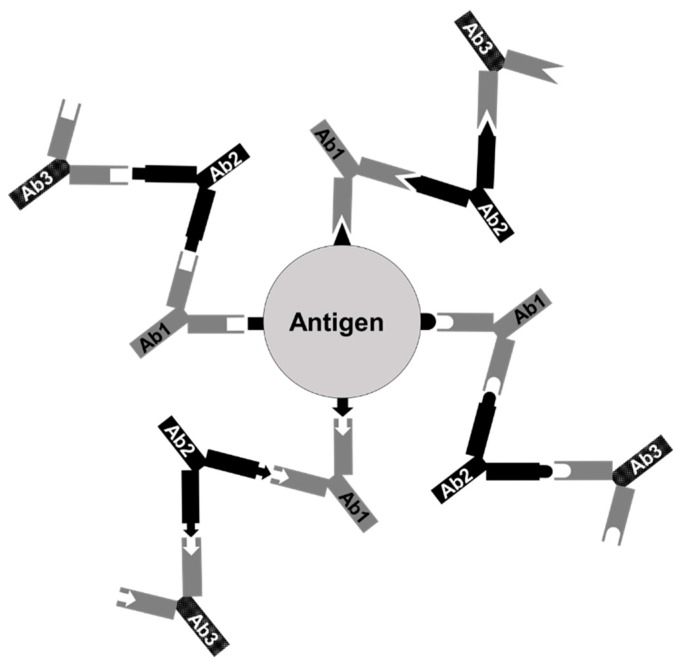
The immunological network theory proposed by Jerne. After an antigen activates the immune system, the first wave of antibodies (Ab1) is induced in response to different specific epitopes of an antigen. The second wave of antibodies (Ab2), anti-Id Abs, is further produced according to the antigen-binding fragments (Fabs, also called idiotypes (Ids)) displayed by Ab1. The Ids of Ab2 may resemble epitopes of the original antigen as internal images and can stimulate the synthesis of anti-anti-idiotype antibodies, Ab3, which principally is of similar specificity to Ab1.

Based on the immunological network theory proposed by Jerne, the interactions between idiotypes (Ids) and anti-idiotype antibodies (anti-Id Abs) have shown an ability to regulate and control the immune system in a stable status [16,17]. Based on the theory (Figure 1), after an antigen enters into the host body, the first wave of antibodies, Ab1, is evoked under the stimulation of immunogenic epitopes of an antigen. Afterwards, according to different antigen-binding fragments (Fabs), also called idiotypes (Ids), displayed by Ab1, the immune system further produces the anti-idiotype antibodies (anti-Id Abs), termed Ab2, which may present different internal images resembling the original epitopes. Likewise, Ab2 can stimulate the production of anti-anti-idiotype antibodies, Ab3, which principally is of similar specificity to Ab1. In other words, anti-Id Abs that express internal images similar to different epitopes of the original antigens may serve as a potential anti-Id vaccine for inducing specific immune responses to the original antigens that they imitate [16,17]. Vaccines based on anti-Id Abs have been so far applied in the control of numerous pathogenic diseases [16,17] and cancers [18]. Therefore, anti-Id Abs showing surrogate immunogenic epitopes have opened an additional area of vaccine development [16,17]. However, anti-Id Abs are so far rarely used as a strategy in the development of fish vaccines. To our knowledge, only two studies on fish anti-Id vaccines have shown effective immunity and protection against fish bacterial pathogens in Japanese flounders (*Paralichthy olivaceus*) [19] and red drum (*Sciaenops ocellatus* L.) [20] induced by intraperitoneal injection with anti-Id Abs. These significant investigations further encouraged us to believe that the anti-Id Abs may fulfill the need for improving immunogenic epitope integrity without the harsh inactivation processes to induce more effective immune responses against *V. harveyi*.

In the present study, we aimed to generate an anti-Id vaccine that would display numerous internal images resembling different epitopes of *V. harveyi* antigens. Anti-*V. harveyi* antibodies from the infected grouper sera were produced, and their Id-containing Fab portion was then injected into rabbits for the production of rabbit anti-Id sera. The rabbit anti-Id IgG with *V. harveyi*-like internal images was collected to prepare its Fab portion, anti-Id IgG (Fab), which was the anti-Id vaccine used for immunizing grouper in the present study. The resulting anti-Id IgG (Fab) was then intraperitoneally injected twice at a 21-day interval into grouper. We examined the ability of anti-Id IgG (Fab) to induce effective anti-*V. harveyi* immune responses, in comparison with the inactivated vaccine consisted of killed *V. harveyi*. Three weeks after the last immunization, protective efficacy in grouper was also evaluated after a lethal peritoneal challenge of 6 × 10^6^ CFU (colony-forming unit) of *V. harveyi* (Vh MML-1).

## 2. Materials and Methods 

### 2.1. Bacterial Strains and Culture 

The taxonomically related local virulent strain (Vh MML-1) of *V. harveyi* was isolated from diseased grouper collected from a fish farm in southern Taiwan. In addition, *V. harveyi* BCRC13812 strain isolated from seawater enriched with glycerol and nitrate was purchased from Bioresource Collection and Research Center (BCRC), Food Industry and Development Institute (Hsinchu, Taiwan). The virulence of Vh MML-1 and BCRC13812 strains were, respectively, determined to be a highly virulent strain and a lowly virulent strain (Figure S1). Bacteria were grown in tryptic soy broth (TSB, Difco) with 2% NaCl at 25 °C for 18 h to mid-logarithmic phase [21].

### 2.2. Animals 

Orange-spotted grouper (*Epinephelus coioides*), weighing 50 ± 8 g, were purchased from a disease-free farm in southern Taiwan. All fish were housed in high containment facilities and were cultivated in 300 L fiberglass-reinforced plastic (FRP) tanks supplied with filtered and aerated regular seawater. Every day fish were fed twice with commercial dry pellets (Hai-Yu, Taiwan). In addition, the fish health status was also monitored every day. After one week, fish (weighing 56 ± 10 g) were ready for experimental use. All administrations to fish were reviewed and approved by the Institutional Animal Care and Use Committee, National Pingtung University of Science and Technology (NPUST-106-049)**.**

### 2.3. Inactivated V. harveyi (Vh MML-1) Bacteria

The highly virulent *V. harveyi* strain (Vh MML-1) was grown on tryptic soy agar (TSA) with 2% NaCl at 25 °C overnight. Single colony of *V. harveyi* was picked up from the agar plate and cultured in 5 ml of TSB with 2% NaCl at 25 °C for 18 h. Afterwards, further expand culture to an OD_600_ of 1 was performed in 500 ml of TSB with 2% NaCl. The bacteria were then inactivated with 0.3% (v/v) of formalin for 24 h and washed three times with PBS to remove formalin. The bacterial pellet was re-suspended in 40 ml of PBS. In order to confirm the inactivation of bacteria, 0.1 mL of the resulting inactivated bacteria suspension was plated on TSA with 2% NaCl and no colony was present on TSA after growth at 25 °C overnight. The inactivated bacteria were then stored at 4 °C until use.

### 2.4. Bacterial Lysate 

The bacterial lysate used in the present study was prepared from the highly virulent Vh MML-1 strain of *V. harveyi,* as described previously [21], with minor modifications. Briefly, *V. harveyi* (Vh MML-1) was cultured in 50 ml of TSB with 2% NaCl at 25 °C to an OD_600_ of 1. The bacteria were collected by centrifugation at 3000× *g* for 10 min and then washed three times with saline. Afterwards, bacteria were re-suspended in 2 mL saline, sonicated by using a VCX 130 ultrasonic processor (Sonics), and then centrifuged at 12,000× *g* for 30 min at 4 °C. The resulting soluble supernatant was used as the *V. harveyi* lysate. The lipopolysaccharide (LPS) in the *V. harveyi* lysate was removed by the Detoxi-Gel Endotoxin Removing column (Thermo Scientific) and its level (below 0.1 EU/mL) was confirmed by the Pierce LAL Chromogenic Endotoxin Quantitation Kit (Thermo Scientific) [22]. The protein concentration of the bacterial lysate was determined by using the dye-binding DC protein assay (Bio-Rad) with bovine serum albumin (BSA) as a standard. Aliquots of the bacterial lysate were stored at −20 °C until use.

### 2.5. Grouper Anti-V. harveyi Antibodies (Ab1) 

As shown in Figure 1, anti-Id Abs (Ab2) are produced from the Ids of Ab1. In order to display more abundant and comprehensive *V. harveyi*-like internal images on anti-Id Abs, *V. harveyi*-infected grouper antibodies that could recognize critical antigens during infection were used as Ab1 to prepare anti-Id Abs. *V. harveyi*-infected grouper sera were collected as described previously, with minor modifications [21]. Briefly, three grouper were intraperitoneally injected with 1 × 10^4^ CFU/100 μl of *V. harveyi* (BCRC13812), an amount 100 times lower than its LD_50_ (1 × 10^6^ CFU), and their sera were collected three weeks later to filter through a 0.45 μm membrane (Millipore). Anti-*V. harveyi* antibodies from the infected grouper sera were purified by Bio-Scale Mini Protein A cartridges (Bio-Rad) according to the previous procedure [23]. The specificity of purified antibodies to *V. harveyi* was then determined by Western blotting. 

### 2.6. Production of the Anti-Id Vaccine, Anti-Id IgG (Fab) 

In order to reduce the isotype influence of Ab1, the purified grouper anti-*V. harveyi* antibodies were digested with papain to obtain their Fab (Id) for preparing anti-Id Abs (Ab2) [24]. Briefly, 5 mg/ml of purified anti-*V. harveyi* antibodies were digested with 1 mg/ml of papain (Sigma) in 15 ml of digestion buffer (20 mM EDTA and 20 mM cysteine in PBS) at 37 °C overnight [24]. The Id purification with Bio-Scale Mini Protein A cartridges (Bio-Rad) was further undertaken and the Id quality was confirmed by SDS-PAGE. New Zealand rabbits purchased from the Livestock Research Institute, Council of Agriculture, Taiwan, were then subcutaneously injected twice at a two-week interval with 1 mg of Id emulsified with Freund’s adjuvant (Sigma) at a 1:1 ratio (v/v). Two weeks after the second injection, rabbit anti-Id sera were collected to further purify their IgG with Bio-Scale Mini Protein A cartridges (Bio-rad). The purified rabbit anti-Id IgG was further digested with papain as described earlier to obtain the Fab portion of anti-Id IgG (anti-Id IgG (Fab)), following an additional Protein A purification. The resulting anti-Id IgG (Fab) was the anti-Id vaccine used in the present study to show the internal images mimicking the epitopes of antigens of *V. harveyi*. Western blot analysis was undertaken to examine the *V. harveyi*-like antigenicity of rabbit anti-Id IgG (Fab) by comparison with normal rabbit IgG (Sigma-Aldrich) and antisera collected from rabbits one month after intravenous infection with 1 × 10^4^ CFU of *E. coli* (*Escherichia coli*). The protein concentration of anti-Id IgG (Fab) was determined by using the dye-binding DC protein assay (Bio-Rad) with bovine serum albumin (BSA) as a standard. Aliquots of anti-Id IgG (Fab) were stored at −20 °C until use.

### 2.7. Immunization in Grouper 

Four groups of 25 fish each were immunized respectively by intraperitoneal injection with anti-Id IgG (Fab) (10 μg/0.1 mL of PBS/fish), inactivated *V. harveyi* bacteria (1 × 10^8^ CFU/0.1 mL of PBS/fish), normal rabbit IgG (10 μg/0.1 ml of PBS/fish), and PBS (0.1 ml/fish) emulsified with the Montanide ISA 763 AVG adjuvant (Seppic, France) in the ratio recommended by the manufacturer. Three weeks later, all animals from different groups were boosted according to the same regimen. During the immunization schedule, the immunoassays, including Western blot, serum titer assay, and lymphocyte proliferation, were performed to evaluate the induced anti-*V. harveyi* immunity in grouper.

### 2.8. Antigenic Specificity of Immunized Grouper Sera 

The antigenic specificity of anti-Id IgG (Fab)-immunized grouper sera was examined by Western blotting three weeks after the second immunization [21]. Briefly, aliquots of *V. harveyi* lysate (20 μg/well) were separated by 12% homologous SDS-PAGE and electrophoretically transferred to a polyvinylidene difluoride membrane (Millipore). After blocking with 5% skim milk in PBS, strips of the membrane were cut and probed with sera from grouper administrated with anti-Id IgG (Fab), inactivated *V. harveyi* bacteria, normal rabbit IgG, or PBS for 1 h at 37 °C. Incubation with *V. harveyi*-infected grouper sera was also conducted. Bound grouper antibodies on strips were detected with 1:1000-diluted guinea pig anti-grouper immunoglobulin sera and then alkaline phosphatase-conjugated, 1:1000-diluted goat anti-guinea pig IgG (Sigma-Aldrich). The subsequent color development was processed, as described previously [21]. 

### 2.9. Grouper Serum Titer Assay 

Following immunization, fish sera were collected every three weeks and their serum titers were examined by ELISA, as described previously [21], with minor modifications. Flat-bottomed 96-well polystyrene microplates (Nunc) were coated with 100 μL/well of *V. harveyi* lysate (10 μg/mL) in 0.1 M carbonate/bicarbonate buffer (pH 9.4) and incubated overnight at 4 °C. Each well was then washed with PBS and blocked with 5% BSA in PBS (blocking buffer). Samples of 1:50 diluted grouper serum in serial dilution were added to the wells (50 μL/well) and incubated for 1.5 h at 37 °C. After three washes with PBST (0.05% Tween 20 in PBS), the wells were incubated with 1:1000-diluted guinea pig anti-grouper immunoglobulin sera for 1 h at 37 °C. PBST washes were carried out again, and each well was incubated with 50 μL of biotinylated goat anti-guinea pig IgG (Vector Laboratories) diluted in the blocking buffer (1:3000) for 1 h at 37 °C. After washing with PBST, 50 μL of streptavidin/peroxidase (1:3000 dilution) was added to each well. After incubation for 1 h at room temperature, color development and serum titer determination were then performed as described previously [21].

### 2.10. Lymphocyte Proliferation Assay 

Since the head kidney is an important lymphoid organ in fish, in the present study, the lymphocyte proliferation to *V. harveyi* lysate in the head kidney of immunized grouper was analyzed to evaluate whether protective cell-mediated immunity was induced. Three weeks after the second immunization, three fish per group were sacrificed to collect their head kidney lymphocytes via 34–51% percoll gradient isolation (Sigma, St. Louis, MO, USA) under sterile conditions [21]. Afterwards, the lymphocytes were cultured in triplicate in 96-well culture plates at a concentration of 2 × 10^5^ cells per well in 200 μl of L-15 culture medium (CM). The cells in each well were stimulated with 20 μg/mL of *V. harveyi* lysate and incubated for 72 h at 25 °C. Con A (10 μg/mL)- and CM-treated cultures were also, respectively, conducted to use as positive and negative controls. The lymphocyte proliferation induced by *V. harveyi* lysate was monitored by using the BrdU (5-bromo-2’-deoxyuridine) Colorimetric Cell Proliferation ELISA Kit (Roche) according to the manufacturer’s instructions [21]. Finally, the stimulation index (stimulation index (SI) = OD_450_ values from *V. harveyi* lysate-treated cultures or Con A-treated cultures/OD_450_ values from CM-treated control cultures) of each group was calculated as described previously and expressed as the mean ± standard deviation (SD).

### 2.11. Bacterial Challenge 

In order to evaluate whether the induced immune responses could protect fish from *V. harveyi* infection, three weeks after the second immunization, four groups of 20 fish each were challenged with an intraperitoneal injection of 6 × 10^6^ CFU of the highly virulent *V. harveyi* (Vh MML-1), an amount 10 times higher than LD_50_ that we had previously determined according to the previous process [25]. After the challenge, fish were observed daily for an additional 28 days and the survival rate in each group was recorded every day [21].

### 2.12. Statistical Analysis 

The data in the present study were statistically analyzed as follows, according to previous studies [21,26,27,28]. Grouper serum titers (Log_10_) were statistically compared using the Nested design and the means at different time points in each group were tested by least significant difference (LSD) multiple comparison. SI values of different immunization groups were statistically compared using one-way ANOVA. The survival rates of different groups were analyzed by the chi-square test. A *p*-value of less than 0.05 was considered to be significant.

## 3. Results

### 3.1. V. harveyi-Like Antigenicity of Rabbit Anti-Id IgG (Fab) 

**Figure 2:**
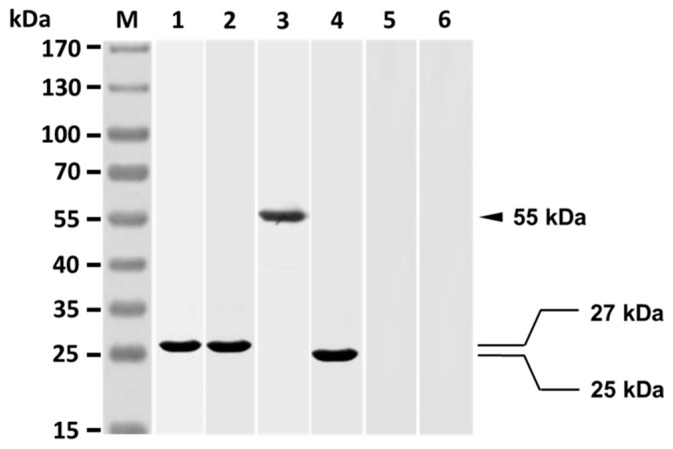
Antigenicity assay of rabbit anti-Id IgG (Fab). The purified Fab of anti-*V. harveyi* antibodies collected from infected grouper was separated by sodium dodecyl sulfate polyacrylamide gel electrophoresis (lane 1) and analyzed with rabbit anti-Id IgG (lane 2). In addition, anti-Id IgG (lane 3) and its Fab portion (lane 4) were, respectively, analyzed with *V. harveyi*-infected grouper sera. Normal rabbit IgG (lane 5) and rabbit anti-*E. coli* sera (lane 6) were also analyzed with *V. harveyi*-infected grouper sera. Standard protein markers are shown to the left (lane M).

SDS-PAGE analysis showed that the resulting Fab of grouper anti-*V. harveyi* antibodies clearly consisted only of a 27 kDa protein (Figure 2, lane 1), which is the expected Fab of the purified grouper antibodies. Western blot analysis revealed that rabbit anti-Id IgG reacted strongly with the Fab (27 kDa) of grouper antibodies (Figure 2, lane 2). The *V. harveyi*-infected grouper sera were found to recognize a 55 kDa protein of rabbit anti-Id IgG (Figure 2, lane 3), which is expected of the heavy chain of rabbit IgG. The finding therefore indicates that the heavy chain of rabbit anti-Id IgG showed *V. harveyi*-like internal images. The *V. harveyi*-infected grouper sera could recognize a 25 kDa protein displayed by the Fab portion of anti-Id IgG (Figure 2, lane 4), while the *V. harveyi*-infected grouper sera could not recognize anything in normal rabbit IgG (Figure 2, lane 5) nor in antisera from rabbits infected with *E. coli* (Figure 2, lane 6). In other words, rabbit anti-Id IgG (Fab) displayed the specific *V. harveyi*-like internal images, but normal rabbit IgG and rabbit anti-*E. coli* sera did not. 

### 3.2. Strong Antibody Responses Elicited by Anti-Id IgG (Fab) in Grouper

**Figure 3:**
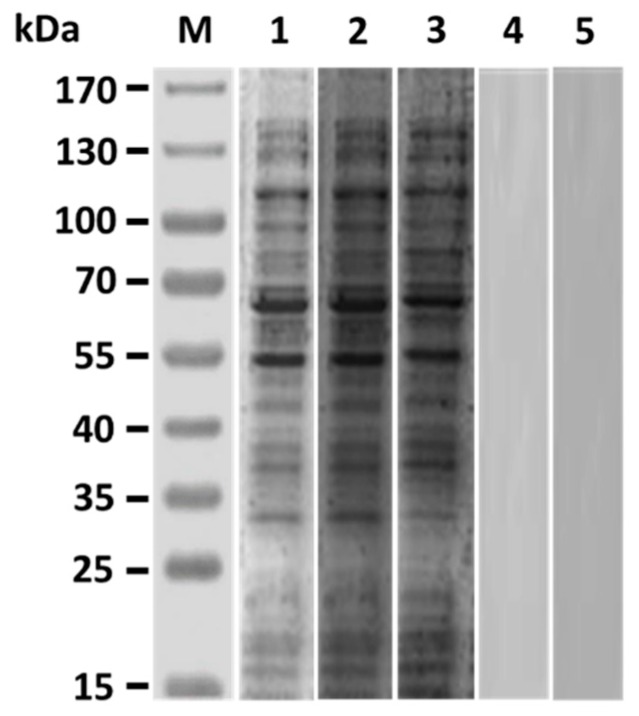
Antigenic specificity of immunized grouper sera. Three weeks after the second immunization, grouper sera from different groups were collected to analyze their antigenic specificity by Western blot. *V. harveyi* lysate was probed with sera from grouper immunized with anti-Id IgG (Fab) (lane 2), inactivated *V. harveyi* bacteria (lane 3), normal rabbit IgG (lane 4), or PBS (lane 5). The *V. harveyi*-infected grouper sera (lane 1) were also conducted as a positive control. Standard protein markers (lane M) are shown at the left.

**Figure 4:**
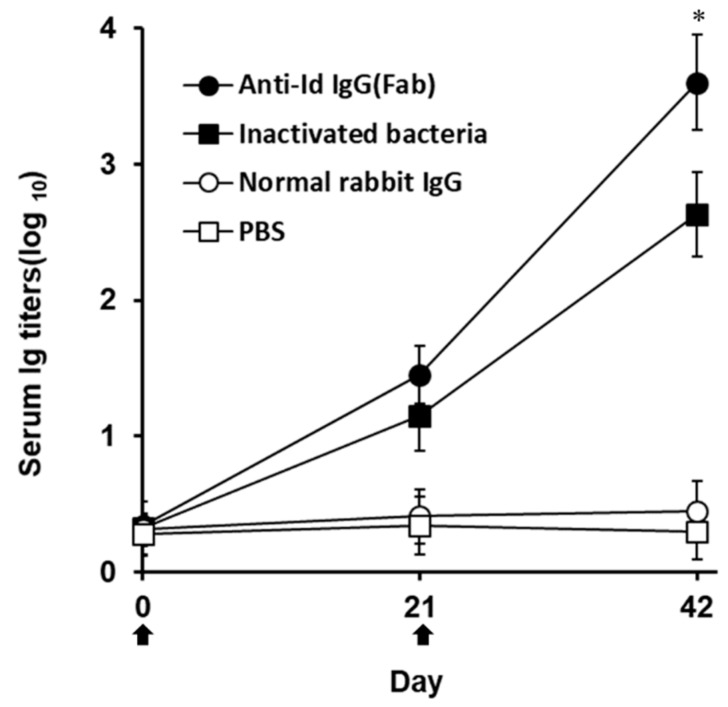
Anti*-V. harveyi* serum titers in immunized grouper. Grouper were intraperitoneally immunized twice (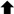) with anti-Id IgG (Fab) (●), inactivated bacteria (■), normal rabbit IgG (○) or PBS (□). Sera were collected from three fish per group on days 0, 21, and 42 and their anti-*V. harveyi* serum titers were determined by ELISA. Results were presented as the mean of log_10_ titers ± SD. * A significant difference (*p* < 0.05) exists when comparing the anti-Id IgG (Fab) group to the inactivated bacteria group.

Western blot studies of grouper immunized sera obtained three weeks after boosting demonstrated that both anti-Id IgG(Fab) and inactivated *V. harveyi* bacteria led to production of serum antibodies against numerous proteins in the lysate of *V. harveyi* (Figure 3, lanes 2 and 3), which were also recognized by *V. harveyi*-infected grouper sera (Figure 3, lane 1). However, sera from grouper immunized with normal rabbit IgG or PBS did not recognize anything in the *V. harveyi* lysate (Figure 3, lanes 4 and 5). Therefore, intraperitoneal immunization with rabbit anti-Id IgG (Fab) in grouper could elicit a specific serum response to the proteins of *V. harveyi*. These results were also consistent with those from Figure 2 and emphasized again that the *V. harveyi*-like antigenicity shown by internal images was really retained in the anti-Id IgG (Fab) produced from rabbits immunized with the Fab of anti-*V. harveyi* grouper antibodies. Three weeks after priming, both anti-Id IgG (Fab) and inactivated bacteria were able to elicit serum titers against *V. harveyi* lysate, but there was no significant difference (*p* > 0.05, Nested design) between the means of these two groups (Figure 4). On the other hand, three weeks after boosting, serum titers induced by anti-Id IgG (Fab) were significantly higher (*p* < 0.05, Nested design) than those induced by inactivated bacteria (Figure 4). During the 6-week serum titer study, however, grouper immunized with normal rabbit IgG or PBS displayed little, if any, anti-*V. harveyi* serum titers (Figure 4). Therefore, anti-Id IgG (Fab) could elicit high levels of anti-*V. harveyi* antibodies, indicating the importance of *V. harveyi*-like internal images shown by anti-Id IgG (Fab). 

### 3.3. High Lymphocyte Proliferation Induced by Anti-Id IgG (Fab) in Grouper 

**Figure 5:**
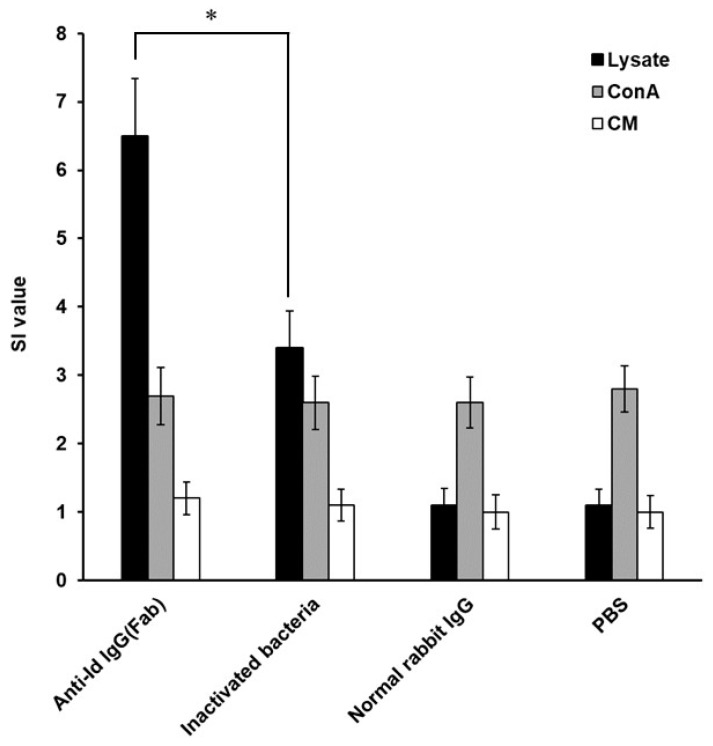
Proliferation responses against *V. harveyi* in immunized grouper. Groups of fish were intraperitoneally immunized twice with anti-Id IgG (Fab), inactivated bacteria, normal rabbit IgG, or PBS. Three weeks after the second immunization, head kidney lymphocytes stimulated with *V. harveyi* lysate (■), Con A (■), or culture medium (CM) (☐) were prepared from three fish per group and their proliferation responses were then analyzed and expressed as stimulation index (SI) values. Results were presented as the mean of SI values ± standard deviation (SD). * A significant difference (*p* < 0.05) exists when comparing the anti-Id IgG (Fab) group to the inactivated bacteria group.

Under the stimulation of *V. harveyi* lysate, the anti-Id IgG (Fb) elicited significantly higher SI values (*p* < 0.05, ANOVA) than inactivated bacteria (Figure 5). However, immunization with normal rabbit IgG or PBS induced little proliferation to *V. harveyi* lysate in fish lymphocytes (Figure 5). As positive and negative controls, lymphocytes from all groups of fish were, respectively, stimulated with T cell mitogen, Con A (10 μg/mL), or culture medium (CM) and were found to proliferate to a similar extent (*p* > 0.05, ANOVA). Therefore, immunization with anti-Id IgG (Fab) in grouper rendered an enhanced lymphocyte proliferation response specific to *V. harveyi* lysate. 

### 3.4. Protection Against V. harveyi Challenge in Grouper 

**Figure 6:**
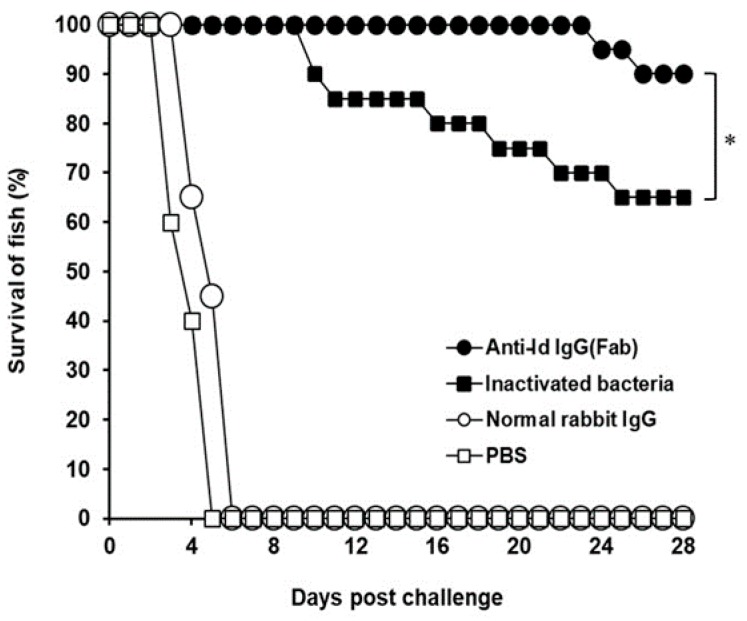
Survival of immunized grouper after a lethal challenge of the virulent strain of *V. harveyi* (Vh MML-1). Groups of fish were intraperitoneally immunized twice with anti-Id IgG (Fab) (●), inactivated bacteria (■), normal rabbit IgG (○), or PBS (□). Three weeks after the second immunization, four groups of 20 fish each were intraperitoneally infected with 6 × 10^6^ CFU (colony-forming unit) of V. harveyi (Vh MML-1). Animals were observed daily for an additional 28-day period and the final survival rates were calculated. * A significant difference (*p* < 0.05) exists when comparing the anti-Id IgG (Fab) group to the inactivated bacteria group.

After challenge, the survival rate in each group was recorded (Figure 6). All fish administrated with normal rabbit IgG or PBS died within 6 days after challenge and showed no protection against the lethal challenge. Thirteen out of 20 fish immunized with inactivated *V. harveyi* bacteria survived during the challenge study and showed a protection of 65%. However, only two fish in the group administrated with anti-Id IgG (Fab) died, respectively, on days 24 and 26 after challenge. Therefore, vaccination with anti-Id IgG (Fab) resulted in a 90% survival rate, which was significantly higher (*p* < 0.05, chi-square test) than that of the inactivated bacteria group. The survival rate increased by 25% in the group of fish immunized with anti-Id IgG (Fab). Thus, vaccination with rabbit anti-Id IgG (Fab) provided a substantial resistance to the experimental challenge of *V. harveyi*. 

## 4. Discussion

Generally, aquatic bacteria like *V. harveyi* produce different critical antigens during infection to aid themselves to infect hosts [11]. Initiation of strong immunity to simultaneously react with epitopes of these antigens would be more likely to alleviate bacterial infection in fish [12]. More effort is therefore needed to develop effective and reliable anti-bacterial vaccines comprising a broad array of immunogenic epitopes of bacterial antigens. In the present study, we successfully employed the interactions between Id and anti-Id Abs (Figure 1) to prepare an anti-Id vaccine, anti-Id IgG (Fab), that presents the internal images similar to immunogenic epitopes of *V. harveyi* antigens (Figure 2). Moreover, the resulting anti-Id IgG (Fab) was demonstrated to be able to induce not only significant *V. harveyi*-specific humoral and cell-mediated immunity (Figure 3), but also high protection (90%) against a lethal experimental challenge of the highly virulent strain of *V. harveyi* (Vh MML-1) (Figure 6). Even though we do not know if the internal images presented by rabbit anti-Id IgG (Fab) were of 100% similarity, we reasonably believe that the *V. harveyi*-like antigenicity of the internal images of anti-Id IgG (Fab) remained high enough since it resulted in subsequent immune responses (Figure 3, Figure 4 and Figure 5) and protection (Figure 6). Unlike anti-Id IgG (Fab), however, inactivated *V. harveyi* bacteria elicited only moderate immunity and protection against *V. harveyi* (Vh MML-1). Therefore, the ability of anti-Id IgG (Fab) to present the internal images that are similar to the epitopes of the original *V. harveyi* antigens is a particularly attractive characteristic. 

Besides the protein antigens, bacteria possess non-protein antigens, such as lipid [29,30] or carbohydrate [31,32,33,34], to influence disease progression and severity. The biosynthesis of carbohydrates or lipids is a complex, multi-enzymatic process [35,36] and not directly template-driven as is the case for proteins, thereby resulting in a challenge to obtain pure, defined carbohydrates and lipids. However, based on previous studies, anti-Id Abs can display internal images that imitate the immunogenic epitopes or conformations of non-protein and protein antigens [16,17]. Therefore, anti-Id Abs can show more abundant and integral antigenicity derived from both protein and non-protein antigens to broaden more effective immune responses [16,17]. Gram-negative bacteria like *V. harveyi* possess epitopes of non-protein antigens made by lipids or carbohydrates, which are very difficult to express by DNA recombination technology. According to the results in the present study, anti-Id IgG (Fab) enhanced significantly stronger immunity (Figure 4 and Figure 5) and protection (Figure 6) in grouper than the inactivated bacteria. These enhanced activities elicited by anti-Id IgG (Fab) appear to be a consequence of their internal images showing more comprehensive epitopes of protein and non-protein antigens of *V. harveyi*. Generally, due to the harsh formalin inactivation process, some essential structural and immunogenic components of microorganisms are denatured to result in inefficient immune responses [15]. Formalin-inactivated *V. harveyi* bacteria used in this study, therefore, could not show more comprehensive epitopes than those displayed by anti-Id IgG (Fab) and thus caused low immunity (Figure 4 and Figure 5) and protection (Figure 6) against the highly virulent *V. harveyi* (Vh MML-1). However, further studies are needed to identify these non-protein epitopes imitated by internal images of anti-Id IgG (Fab).

An indicative hallmark of an efficacious vaccine used in fish is the ability to induce both strong humoral and cell-mediated immunity [37,38]. In the present study, enhanced anti-*V. harveyi* titers detected in the grouper sera (Figure 3 and Figure 4) following peritoneal immunization with anti-Id IgG (Fab) pointed out that B cell-mediated humoral immune response should contribute to the resistance against the highly virulent *V. harveyi* (Vh MML-1). In addition, serum titers induced by anti-Id IgG (Fab) were significantly higher (*p* < 0.05, Nested design) than those induced by inactivated bacteria (Figure 4). Beside humoral immunity, after peritoneal immunization in grouper, we also concentrated much attention on the lymphocyte proliferation response, an activity that has been demonstrated to positively correlate with cell-mediated immunity in our previous study [21]. Three weeks after boosting, an increased lymphocyte proliferation response to the lysate proteins of the virulent *V. harveyi* (Vh MML-1) was readily observed in grouper immunized with anti-Id IgG (Fab) (Figure 5). Furthermore, the anti-Id IgG (Fb) elicited significantly higher SI values (*p* < 0.05, ANOVA) than inactivated bacteria (Figure 5). Based on these results from the anti-*V. harveyi* immunoassays, anti-Id IgG (Fab) displays crucial internal images similar to both B- and T-cell epitopes of *V. harveyi* antigens and is better than inactivated bacteria in eliciting significant *V. harveyi*-specific mixed Th1/Th2 immune responses in grouper.

Previous studies have indicated that antigenic variation, depending on different strains and/or isolates of *V. harveyi,* results in a difficulty in developing a cross-protective vaccine against *V. harveyi* [39]. More importantly, in the present study, both cell-mediated and humoral immune responses induced by anti-Id IgG(Fab) protected 90% of grouper against a lethal challenge caused by the heterologous, highly virulent Vh MML-1 strain of *V. harveyi* and allowed fish to survive for a long period of 28 days after the experimental challenge (Figure 6). Thus, results from our challenge study suggest that the anti-Id vaccine, ant-Id IgG (Fab), developed from grouper antibodies against the lowly virulent strain in this study can induce effective cross-protection against the highly virulent strain. In further comparison, anti-Id IgG (Fab) elicited a significantly higher protective rate (90%) in grouper than inactivated bacteria (65%). Therefore, immunity enhanced by anti-Id IgG (Fab) provided a substantial resistance to the experimental challenge of the highly virulent strain of *V. harveyi* (Vh MML-1). A significant study proposed by Yongjuan et al. has shown protection (75~85%) against *Vibrio anguillarum* infection in Japanese flounders induced by intraperitoneal administration with monoclonal anti-idiotype antibody [19]. In addition, a high relative percentage survival (86.4~88.1%) against *Edwardsiella tarda* was also found in red drum injected intraperitoneally with a single chain variable fragment (scFv) vaccine derived from an anti-idiotype antibody [20]. Despite the differences in fish species and pathogenic bacteria used for in vivo studies, the notable vaccine potency (survival rate/protection) observed in the present study and those recorded by others certainly indicate that the administration of anti-Id vaccines in fish conferred substantial immunity in improving protection against aquatic pathogens. Thus, the anti-Id vaccine, anti-Id IgG (Fab), prepared in the present study could be applied in developing an efficacious vaccine against *V. harveyi* for future farm use in fish.

## 5. Conclusions

We successfully produced an anti-Id vaccine, anti-Id IgG (Fab), displaying internal images that are similar to epitopes of *V. harveyi* antigens. In addition, anti-Id IgG (Fab) administered in the grouper peritoneal cavity enhances *V. harveyi*-specific humoral and cell-mediated immune responses to cross-protect grouper from the challenge of the heterologous, highly virulent strain of *V. harveyi*. The capability of this anti-Id IgG (Fab) to imitate the epitopes of *V. harveyi* antigens and effectively induce protective immunity would be advantageous for its application in developing an efficacious vaccine against *V. harveyi* for future farm use in fish.

## Supplementary Materials

The following are available online at https://www.mdpi.com/2076-393X/7/4/210/s1: Figure S1: The virulence of the *V. harveyi* isolates, Vh MML-1 and BCRC13812, was evaluated by challenging grouper for 28 days. Three groups of 20 fish each were challenged (infected) by intraperitoneal injection with the Vh MML-1 strain (1.2 × 10^6^ CFU/0.1 mL PBS/fish), the BCRC13812 strain (1.2 × 10^6^ CFU/0.1 mL PBS/fish), or PBS (0.1 mL PBS/fish). Afterwards, fish were observed daily for 28 days and deaths were recorded as they occurred. Then, the survival rate in each group was calculated. The control fish administrated with PBS showed no clinical signs or mortality during the period. In contrast, all fish injected with the Vh MML-1 strain died in 11 days and showed 100% mortality. By comparison, fish injected with the BCRC13812 strain displayed 40% mortality by day 28 post-infection. The Vh MML-1 strain led to significantly higher mortality in grouper than the BCRC13812 strain (*p* < 0.05, chi-square test). Thus, the virulence of Vh MML-1 and BCRC13812 strains were, respectively, determined to be a highly virulent strain and a lowly virulent strain.
