# Supplementary material for: Anti-Idiotype Vaccine Provides Protective Immunity Against Vibrio Harveyi in Grouper (Epinephelus Coioides)
Source: Vaccines (Basel). 2019 Dec 9;7(4):210. doi: 10.3390/vaccines7040210 (PMC6963308; doi:10.3390/vaccines7040210)
Supplement: Supplementary file 1 [file vaccines-07-00210-s001.pdf]

vaccines

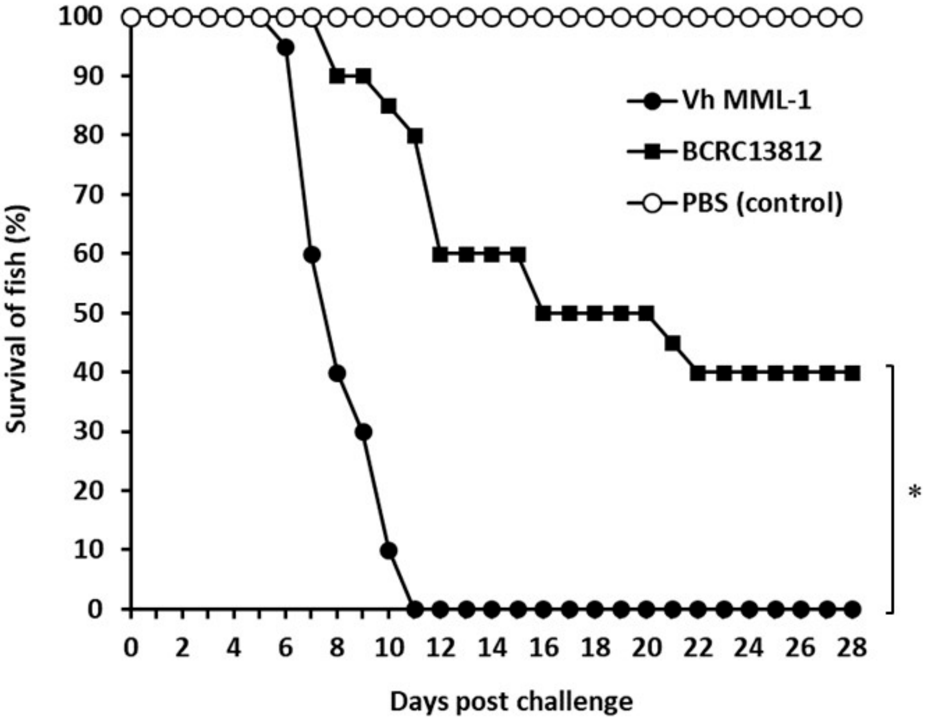

Figure 1. The virulence of the *V. harveyi* isolates, Vh MML-1 and BCRC13812, was evaluated by challenging grouper for 28 days. Three groups of 20 fish each were challenged (infected) by intraperitoneal injection with the Vh MML-1 strain (1.2×10^6 CFU/0.1 ml PBS/fish), the BCRC13812 strain (1.2×10^6 CFU/0.1 mL PBS/fish) or PBS (0.1 mL PBS/fish). Afterwards, fish were observed daily for 28 days and deaths were recorded as they occurred. Then, the survival rate in each group was calculated. The control fish administrated with PBS showed no clinical signs or mortality during the period. In contrast, all fish injected with the Vh MML-1 strain died in 11 days and showed 100% mortality. By comparison, fish injected with the BCRC13812 strain displayed 40% mortality by day 28 post-infection. The Vh MML-1 strain led to significantly higher mortality in grouper than the BCRC13812 strain ($P < 0.05$, Chi-square test). Thus, the virulence of Vh MML-1 and BCRC13812 strains had been respectively determined to be a highly virulent strain and a lowly virulent strain.
